# Reliability and validity of pendulum test measures of spasticity obtained with the Polhemus tracking system from patients with chronic stroke

**DOI:** 10.1186/1743-0003-6-30

**Published:** 2009-07-30

**Authors:** Richard W Bohannon, Steven Harrison, Jeffrey Kinsella-Shaw

**Affiliations:** 1 Department of Physical Therapy, Neag School of Education, University of Connecticut, Storrs, USA

## Abstract

**Background:**

Spasticity is a common impairment accompanying stroke. Spasticity of the quadriceps femoris muscle can be quantified using the pendulum test. The measurement properties of pendular kinematics captured using a magnetic tracking system has not been studied among patients who have experienced a stroke. Therefore, this study describes the test-retest reliability and known groups and convergent validity of the pendulum test measures obtained with the Polhemus tracking system.

**Methods:**

Eight patients with chronic stroke underwent pendulum tests with their affected and unaffected lower limbs, with and without the addition of a 2.2 kg cuff weight at the ankle, using the Polhemus magnetic tracking system. Also measured bilaterally were knee resting angles, Ashworth scores (grades 0–4) of quadriceps femoris muscles, patellar tendon (knee jerk) reflexes (grades 0–4), and isometric knee extension force.

**Results:**

Three measures obtained from pendular traces of the affected side were reliable (intraclass correlation coefficient ≥ .844). Known groups validity was confirmed by demonstration of a significant difference in the measurements between sides. Convergent validity was supported by correlations ≥ .57 between pendulum test measures and other measures reflective of spasticity.

**Conclusion:**

Pendulum test measures obtained with the Polhemus tracking system from the affected side of patients with stroke have good test-retest reliability and both known groups and convergent validity.

## Introduction

Although controversy surrounds the definition of spasticity, Lance described it as "a motor disorder characterized by a velocity dependent increase in tonic stretch reflexes (muscle tone) and increased tendon jerks resulting from disinhibition of the stretch reflex, as one component of the upper motor neuron lesion"[1]. Several tests have been used for quantifying spasticity among patients with diverse sources of upper motor neuron lesions, including stroke, multiple sclerosis, spinal cord injury, and cerebral palsy. Among such tests, the Ashworth [2] or Modified Ashworth [3] are probably used most often. An alternative to these tests' ordinal scales are the real number descriptions of the knee extensors' response to stretch provided by the pendulum test. First described by Wartenberg, the pendulum test depicts movement of the leg following its drop from a horizontal position while subjects are instructed to relax [4]. Since Waltenberg's initial publication, electrogoniometry [5-7], videography [7,8], and magnetic sensing devices [9] have been used to characterize movement of the leg while it oscillates after being dropped. Magnetic sensing devices are the most recent technology to be employed, but their use with patients who have experienced a stroke has been limited to date. We were unable to identify any research that examined the use of magnetic tracking systems during pendulum tests of both lower limbs of patients following stroke. Moreover, we failed to find any literature addressing the performance of the test with a weighted lower limb. Before the pendulum test performed in conjunction with magnetic sensing devices can be recommended for use, it must be shown to have acceptable measurement properties. We therefore purposed to determine the intrasession reliability and validity of the test performed with an unweighted and weighted limb.

## Methods

### Subjects

Based on published information and our expectation that the angle of first reversal of the pendulum test would differ by more than 20 degrees between the more and less involved sides, our analysis suggested that a power of 80% could be achieved with p < .05 with 8 subjects. Therefore, after obtaining written informed consent, we enrolled 8 middle-aged and older, independently ambulatory individuals who had experienced a stroke more than 6 months before. The side of predominate weakness was the right for 5 subjects and the left for 3 subjects. Five subjects were women and 3 were men.

### Procedures

Prior to performing the pendulum tests, several variables were measured to describe the subjects and their neurologic status. These included demographics (age, height, weight, and time since stroke), sensory appreciation of touch with a 5.07 monofilament on both distal lower limbs and feet, comfortable gait speed over a 3 meter distance, resting angles of both knees while subjects were supine with their legs hanging freely off the end of a test table, Ashworth scores (grades 0–4) of bilateral quadriceps femoris muscles, patellar tendon (knee jerk) reflexes (grades 0–4) of both lower limbs, and isometric knee extension force (Newtons) of each lower limb while subjects were seated with their legs vertical.

**Figure 1:**
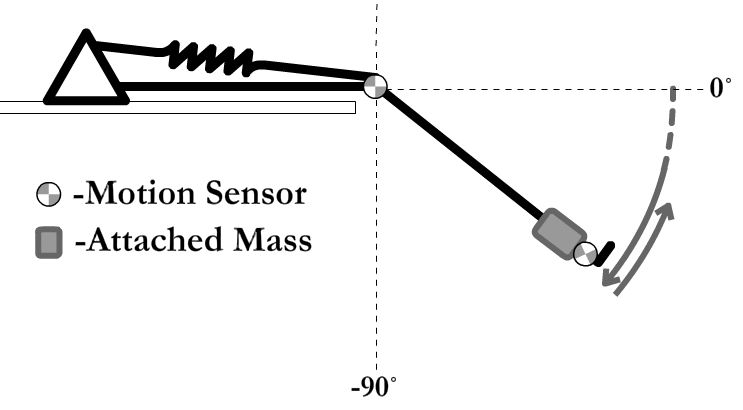
**Line drawing illustrating pendulum test performed with subject supine and leg swinging freely with motion sensors attached and weight secured at ankle**.

**Figure 2:**
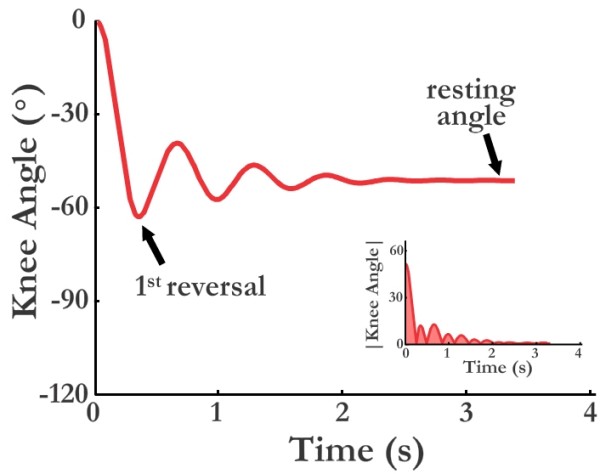
**Representative pendulum test tracing with angle of first reversal identified (large trace) and area under curve highlighted (small insert)**.

The pendulum test was conducted while subjects were supine on a padded table with their nontested leg supported. The test employed the Polhemus Liberty magnetic position tracking system. Two sensors were positioned on the tested lower limb, one laterally over the knee axis of rotation and one just distal to the lateral malleolus (Figure 1). The system transmitter was placed in close proximity on the floor. After checking calibration of the field sensors, the tested leg was passively elevated to horizontal by one of the investigators. Once relaxation was assured by palpation of the patellar tendon, free mobilization of the patella and slight hefts and releases of the leg, data capture was initiated and the leg was dropped. Data capture ended when the leg ceased swinging. This procedure was completed, in random order, twice without a cuff weight at the ankle and twice with a cuff weight (5 lb/2.27 kg) at the ankle. After all subjects were tested, files were imported to Matlab for characterization of pendular kinematics. Characterization consisted of three measures (Figure 2): 1) First angle of reversal, when leg motion first switched from flexion to extension, 2) Area under the curve, the area between the knee angle during oscillations and resting angle, and 3) Velocity to first reversal, the change in knee angle between starting position and first reversal divided by time to first reversal.

### Data analysis

The Statistical Package for Social Sciences was used for all analysis. Summary statistics were calculated. These involved medians and minimum to maximum values for most measurements as the sample sizes were small, they were ordinal in nature, or they not normally distributed. Measurements derived from pendulum tests were an exception. Reliability of the pendulum test measures was established by the intraclass correlation coefficient (ICC). Two aspects of validity were examined statistically: known groups and convergent. Known groups validity entailed using Wilcoxon tests to determine if the pendulum test measures differed significantly between the uninvolved and involved side of the subjects. Convergent validity required the calculation of Spearman correlations (r_s_) to determine if significant relationships existed between the pendulum test measures and other relevant measures of motor status of the involved side.

## Results

Subjects were all either middle-age or older adults. Their height and weight are reported along with other descriptive information in Table 1. Only one subject failed to appreciate touch with the 5.07 monofilament and for that subject, the impairment was limited to the affected lower limb. All subjects walked at less than a meter per second. All had resting knee angles of less than 90 degrees. No subject's unaffected quadriceps demonstrated increased tone (resistance to stretch) during Ashworth testing, but most had some increase in tone on the affected side. Most had knee jerks that were below normal (2/4) on the unaffected side but above normal on the affected side. The subjects' median knee extension force on the affected side was 74.1 percent of that on the unaffected side.

**Table 1:** Summary statistics describing subjects and measurements (excluding pendulum tests) obtained from them.

Variable	Median	Min-Max
Age (yr)	65.5	46–83

Height (cm)	159.7	154.3–171.4

Weight (kg)	63.9	49.5–93.6

Gait speed (cm/sec)	84.0	21.9–97.2

Resting knee angle: affected side (degrees)	65.0	24.0–74.0

Resting knee angle: unaffected side (degrees)	65.5	56.0–72.0

Ashworth grade-quadriceps: affected side (0–4)	1.5	0–3

Ashworth grade-quadriceps: unaffected side (0–4)	0	0–0

Knee jerk: affected side (0–4)	2.5	2–4

Knee jerk: unaffected side (0–4)	1.0	0–4

Knee extension force: affected side (Newtons)	202.8	93.4–400.2

Knee extension force: unaffected side (Newtons)	273.5	169.0–591.4

**Figure 3:**
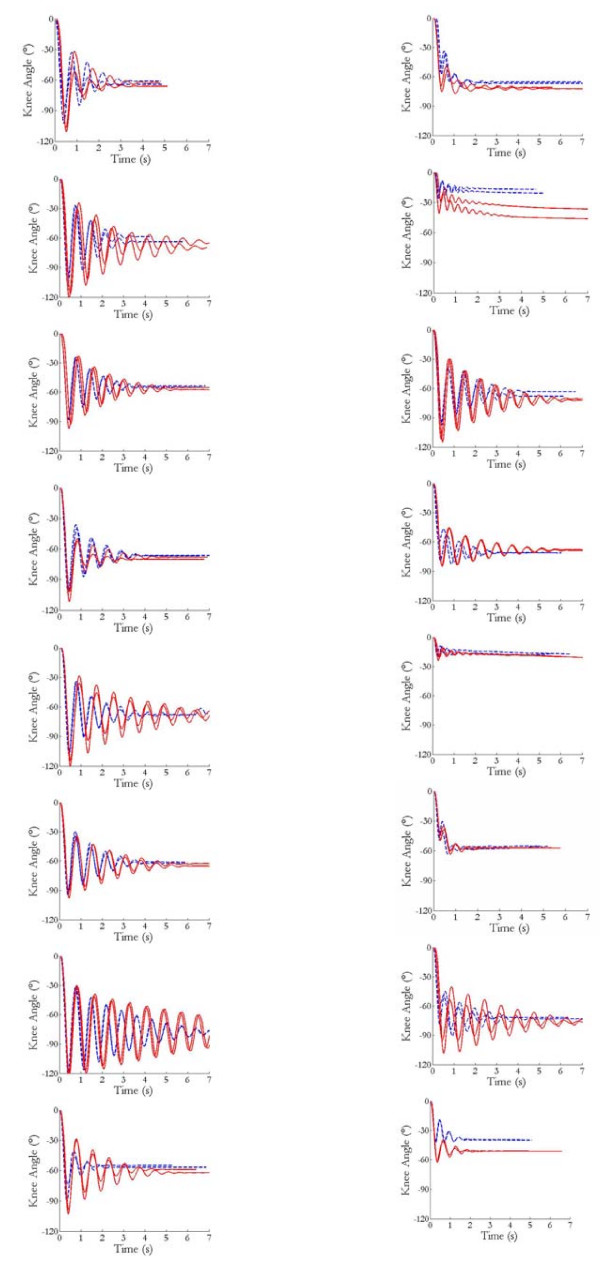
**Pendulum test traces from all subjects**. Those on the left are from the unaffected side while those on the right are from the affected side. Blue dashed lines represent traces obtained without weights whereas red solid lines represent traces obtained with weights.

Pendulum test traces for all subjects are presented in Figure 3. Table 2 summarizes the ICCs associated with the various pendulum test measures obtained from the traces. With the exception of the ICC associated with the velocity to first reversal during testing with no weight on the unaffected side (ICC = .212), all ICCs (.651 to .844) were significant (p < .05). Table 3 compares the pendulum test measures between the affected and unaffected sides. Supporting the known groups validity of the measures, all differed significantly between sides whether or not the test was supplemented with a weight. Convergent validity of the pendulum test measures was affirmed by their strong correlations (.81 to .99) with one another (Table 4). Their validity is further supported by their good correlations with most other motor measures (Table 4). On the involved side, lesser angles of first reversal, smaller areas under the curve, and slower velocities to first reversal were all associated with smaller resting angles (r_s _= .57 to .71), higher Ashworth scores (r_s _= -.63 to -.89), greater knee jerks (r_s _= -.64 to -.79), and lower knee extension forces (r_s _= .59 to .76). However, only the angles of first reversal were significantly correlated (r_s _= .71 to -.80, p ≤ ,01) with all of these measures. None of the pendulum test measures correlated significantly (r_s _= .27 to .57) with gait speed.

**Table 2:** Intraclass correlation coefficients (ICCs) for repeated pendulum test measures

Measure	ICC (p)	95% Confidence Interval	
		Lower	Upper
First Reversal (no weight): affected side	.956 (.001)	.798	.991

First Reversal (weight): affected side	.974 (.001)	.876	.995

First Reversal (no weight): unaffected side	.630 (.034)	-.063	.913

First Reversal (no weight): unaffected side	.863 (.001)	.463	.971

Area under curve (no weight): affected side	.920 (.001)	.656	.983

Area under curve (weight): affected side	.871 (.001)	.487	.973

Area under curve (no weight): unaffected side	.933 (.001)	.705	.986

Area under curve (weight): unaffected side	.976 (.001)	.886	.995

Velocity to first reversal (no weight): affected side	.957 (.001)	.804	.991

Velocity to first reversal (weight): affected side	.844 (.001)	.405	.967

Velocity to first reversal (no weight): unaffected side	.212 (.292)	-.592	.770

Velocity to first reversal (weight): unaffected side	.651 (.029)	-.027	.919

**Table 3:** Paired comparisons relevant to known groups validity of the pendulum test

Measures	Affected Mean (SD)	Unaffected Mean (SD)	Wilcoxon Test	
			Z	(p)
First Reversal-No Weight (degrees)	54.7 (25.4)	99.4 (12.8)	-2.383	(.017)

First Reversal-Weight (degrees)	70.5 (30.9)	109.9 (9.5)	-2.383	(.017)

Area Under Curve-No Weight (degrees*seconds)	24.0 (14.3)	45.4 (23.7)	-2.100	(.036)

Area Under Curve-Weight (degrees*seconds)	37.1 (26.0)	73.2 (46.5)	-1.960	(.050)

Velocity to First Reversal-No Weight (degrees/second)	164.5 (56.5)	240.1 (27.5)	-2.240	(.025)

Velocity to First Reversal-Weight (degrees/second)	173.4 (54.7)	233.6 (27.1)	-1.960	(.050)

**Table 4:** Spearman correlations (probabilities) relevant to the convergent validity of the pendulum test

Measures	First Reversal-NW	First Reversal-W	AUC-NW	AUC-W	Velocity-NW	Velocity-W
First Reversal-W	.99 (.001)					

AUC-NW	.96 (.001)	.96 (.001)				

AUC-W	.91 (.002)	.91 (.002)	.91 (.002)			

Velocity-NW	.96 (.001)	.96 (.001)	.86 (.007)	.81 (.015)		

Velocity-W	.96 (.001)	.96 (.001)	.87 (.005)	.92 (.001)		

Rest Angle	.71 (.050)	.71 (.050)	.67 (.071)	.57 (.139)	.62 (.102)	.64 (.091)

Ashworth	-.80 (.018)	-.80 (.018)	-.73 (.040)	-.63 (.094)	-.85 (.007)	-.89 (.003)

Knee Jerk	-.76 (.028)	-.76 (.028)	-.79 (.019)	-.67 (.070)	-.64 (.086)	-.76 (.028)

Knee Force	.73 (.040)	.73 (.040)	.76 (.028)	.57 (.139)	.67 (.071)	.59 (.126)

Gait Speed	.49 (.217)	.49 (.217)	.57 (.139)	.45 (.260)	.31 (.456)	.27 (.509)

*AUC = area under curve, W = weight, NW = no weight

## Discussion

This study demonstrated that a magnetic tracking system can be used to characterize the pendular kinematics of the leg and thus quantify spasticity of the quadriceps femoris muscles of patients with stroke. By conducting repeated pendulum tests on both the affected and unaffected sides and by employing other tests meant to reflect spasticity, we were able to examine the reliability and validity of the test as well.

Measurements obtained with the magnetic tracking system, like those acquired previously using electrogoniometer and video based systems [6,8,10,11], tended to be reliable. Exceptions were 3 measurements from the unaffected side, which is less likely to be the target of clinical testing anyway. Whether the measurements are reliable across longer periods of time (eg days) and how much of a change would be required to assume a real difference remains to be determined.

The measurements obtained in this study were found to possess both known groups and convergent validity. The known groups in this study were the affected and unaffected sides of the patients with stroke. Unlike Fowler et al [12], we found a difference in angle of first reversal and velocity between sides. Whether measurements obtained from the patients differ from those of control subjects, as they did in the study of Fowler et al, is unknown as we did not have a control group.

If the different measures obtained from the pendulum test (ie, angle of first reversal, area under the curve, velocity) are measuring the same underlying variable, they would be expected to converge (correlate highly), which they did. Moreover, if that variable is spasticity, meaningful correlations between the pendulum test measures and other tests reflective of spasticity (eg, Ashworth) might be anticipated. Based on Feinstein's characterization of correlations as quite good in medical research if they surpass .50 [13], we interpret the correlations between measures of spasticity in our study to be meaningful even if all were not significant. The correlation between pendulum test and Ashworth scale grades supports previous work of Leslie et al who reported a correlation of -.878 between a pendulum test measure and Ashworth grades for 14 patients with multiple sclerosis [14]. The correlations between pendulum test measures and gait speed in our study fell below the .50 criterion of Feinstein. Thus, while the pendulum test is supported as a measure of impairment, its ability to explain an activity limitation such as gait speed was more limited. Although Francis et al have argued that a reduction in spasticity can result in improved function [15], the meta-analysis leading to their conclusion focused on the upper limb. Studies using the pendulum test and other measures to document spasticity in the lower limbs have not shown it to explain reductions in gait speed [11,16,17].

While the objectivity, reliability, and validity of the pendulum test and the simplicity and portability of the Polhemus tracking system provide support for its use, the convergent validity of the measures obtained with the system suggests little advantage of the area under the curve and velocity measures over the angle of first reversal measure. That angle, also called the "first swing excursion," was described by Fowler et al as the "most sensitive outcome measure" in persons with cerebral palsy [18]. There are other measures that can be obtained from pendulum test traces (eg, number of oscillations, angle of first reversal with reference to resting angle) [10,18] that were not derived or tested in our study. Their measurement properties may or may not be comparable to those of the measures tested. As expected, adding weight to the ankle during the pendulum test altered pendular kinematics. Presumably it contributed to the inertia of the leg against which the quadriceps and spasticity served as a brake. Still, the addition of the weight did not improve the measurement properties of the pendulum test and can therefore be judged unnecessary.

## Conclusion

Pendulum test measures obtained with the Polhemus tracking system from the affected side of patients with stroke have good test-retest reliability. The measures also demonstrate both known groups and convergent validity.

## List of abbreviations

ICC: intraclass correlation coefficient; r_s_: Spearman rho correlation; lb: pound; kg: kilogram.

## Competing interests

The authors declare that they have no competing interests.

## Authors' contributions

RWB conceived of the study, contributed to its design, recruited subjects, tested subjects, conducted data analysis, and wrote the original draft of the manuscript. SH contributed to the design of the study, helped to test subjects, generated graphics, and helped to write the manuscript. JK-S recruited subjects, helped to test subjects, and helped to write the manuscript. All authors read and approved the final manuscript.
